# Assessing Cognitive Skills in Early Childhood Education Using a Bilingual Early Language Learner Assessment Tool

**DOI:** 10.3390/jintelligence11070143

**Published:** 2023-07-17

**Authors:** Mei Tan, Hechmi Kilani, Ilia Markov, Sascha Hein, Elena L. Grigorenko

**Affiliations:** 1 HIV Center for Clinical and Behavioral Studies, Columbia University, 722 West 168th Street, New York, NY 10032, USA; mtt2114@cumc.columbia.edu; 2 Texas Institute of Measurement, Evaluation and Statistics, University of Houston, 4349 Martin Luther King Boulevard, Houston, TX 77204, USA; hechmi.kilani@times.uh.edu (H.K.);; 3 Department of Education and Psychology, Free University of Berlin, Fabeckstraße 35, 14195 Berlin, Germany; 4 Center for Cognitive Sciences, Sirius University of Science and Technology, 354340 Sochi, Russia

**Keywords:** kindergarten readiness, tablet-based assessment, 21st century skills

## Abstract

In this article, we propose that basic cognitive skills may be fostered and assessed in early childhood educational (pre-K) settings using a technology-based approach to assessment. BELLA (Bilingual English Language Learner Assessment), designed for use with both monolingual (English or Spanish speaking) and bilingual (English and Spanish speaking) children, is designed to attend to cognitive skill development in addition to (pre-)academic knowledge. Specifically, BELLA assesses analytical, creative, and practical thinking in 3–5-year-old children through unique item content and delivery. BELLA is among the first tablet-based pre-K assessments designed to assess cognitive skills needed for the era of the Anthropocene.

## 1. Introduction

It has been stated that we may be living in a new geologic era of the Earth’s history, the Anthropocene, particularly marked by human-induced effects on the Earth and its larger environment (Crutzen 2002; Lewis and Maslin 2015; Zalasiewicz et al. 2020). What this has come to mean, in practical terms, is a rapidly changing environment, further complicated by economic and social challenges brought about by an increasingly financially and technologically interconnected world (OECD 2018). These are manifested, for example, in issues surrounding the depletion of natural resources, the recognition of the necessity of shared economies, and the rich, growing cultural diversity within communities and countries alike (OECD 2018). To successfully cope with the many new, unforeseen, multi-faceted, and dynamic problems that have ushered in this new era, it has been argued that education must broaden its attention to fostering skills necessary to meet complexity and uncertainty (OECD 2018). Across definitions of skills crucial for facing the 21st century, educational researchers, in line with the Organisation for Economic Cooperation and Development’s (OECD) Future of Education and Skills 2030 (Ananiadou and Claro 2009), agree on a constellation of cognitive, interpersonal and intrapersonal skills that include critical thinking, creative thinking, empathy, and, importantly, the ability to apply skills and knowledge in unfamiliar, fast-evolving conditions (Geisinger 2016; Metz 2011). Assessment, in its turn, must play a role in this shift of education toward broad conceptions of skills and abilities; it has been suggested that rich digital environments may be leveraged to best capture some of these skills (Care et al. 2018; Soland et al. 2013). Most assessments devised thus far to capture these 21st century skills have been designed for children in the primary grades and up. A key question in the field of early childhood development is how and when these skills may be detected and fostered, and whether they can be supported in early childhood, pre-K classrooms, where they have not previously been emphasized in formal assessment.

To illustrate a technology-based approach to encourage cognitive skill development and teachers’ awareness of these skills as early as pre-Kindergarten (pre-K), we describe a new tablet-based assessment specifically designed to support the development of analytical, creative, and practical skills in young children. BELLA (Bilingual English Language Learner Assessment) addresses 3–5-year-old children’s cognitive skill development through unique item content and delivery. To contextualize BELLA, we outline areas of skill development emphasized in pre-K environments today and describe the capabilities currently prioritized in commonly used kindergarten-readiness assessments. We then discuss some of the cognitive skills currently less emphasized in formal preschool assessment and illustrate how they are cultivated through BELLA’s digital environment.

## 2. The Current Focus of Skill Development in Pre-K

### 2.1. Developmental Emphases in Preschool Curricula

Currently in the United States, the central domains tracked to gauge school readiness in pre-K children cover five domains: (1) physical well-being and motor development; (2) social and emotional development; (3) language and literacy development; (4) cognition and general knowledge; and (5) approaches to learning (OHS 2022). These were originally outlined by the National Education Goals Panel in 1991 as foundational for children’s later learning in school. They exemplify the prioritization of children’s general physical and emotional well-being, communication skills, ability to think and acquire knowledge, and attitudes and habits for learning (e.g., self-control, task persistence, attention) in classroom settings (Pan et al. 2019). The consideration of cognitive skills as important outcomes at this developmental and educational stage is limited, primarily focusing on a narrow range of skills such as memory, attention, and executive functioning (Weiland and Yoshikawa 2013; Yoshikawa et al. 2013). Pre-K curricula, however, tend to be comprehensive, addressing multiple aspects of child development.

Many government grant-funded programs, including Head Start, are required to use a “whole-child” curriculum designed to address children’s development across several domains. Whole-child curricula are based on constructivist, experienced-based notions of learning and are considered to be child-centered. They support children’s learning by providing opportunities for them to interact independently with equipment, materials, and other children within the classroom setting in various enrichment activities (Michael-Luna and Heimer 2012; Nguyen et al. 2019). According to the National Survey of Early Child Care and Education carried out in 2012 in the US (NSECE 2012), over 40% of providers then used a whole-child curriculum, and about 34% used a locally developed (by a teacher, school, or district) curriculum or no curriculum at all (Jenkins et al. 2018). The most widely used whole-child curriculum in preschool and Head Start classrooms, according to a national survey, is Creative Curriculum (Jenkins et al. 2018).

Creative Curriculum addresses four domains of development: social-emotional, physical, cognitive, and language. It also attends to seven areas of content knowledge: literacy, mathematics, science, social studies, the arts, technology, and process skills (Copple and Bredekamp 2009; Michael-Luna and Heimer 2012). One of the primary modes of children’s learning in this curriculum is through purposeful play or play-based learning (Taylor and Boyer 2020), which may take place within various areas of interest for children, such as blocks, dramatic play, toys and games, art, cooking, computers, and the outdoors; however, large- and small-group activities facilitated by a teacher also occur daily. Thus, Creative Curriculum, and whole-child approaches in general, are intended to be flexible to allow for varying balances of teacher-directed and child-initiated learning that accommodate the children’s individual differences (Michael-Luna and Heimer 2012). Despite the intentions of such open, play-based curricula to support child development broadly and comprehensively—Creative Curriculum advocates naturalistic formative assessment as well as observation (Dodge et al. 2002)—their original intent is challenged by the growing importance of outcome-based assessments, particularly those in content areas such as math and language. Site-based evaluations of the Creative Curriculum by the National Center for Education Research reflected a lack of short-term gains in children’s reading, phonological awareness, and oral language (Michael-Luna and Heimer 2012; NCER 2008; Zigler et al. 2004). Cognitive-skill development is supported implicitly in such curricula yet is not targeted explicitly in typically used outcome-based assessments. Thus, while early childhood curricula are generally designed to foster a broad range of cognitive skills, including creativity, measuring academic outcomes to gauge children’s progress is often prioritized (Yun et al. 2021).

### 2.2. The Focus of Current Kindergarten Entry Assessments

The aims and methods of assessment have transformed over the years at all levels of education in response to various theoretical developments, practical innovations, and government policies. For example, just three decades ago, the main purposes of assessment were an objective measurement of performance and the predictive sorting or classifying of children for certain occupations or stations in life (Conner 1991; Satterly 1989). In 2001, the National Research Council’s Committee on the Foundations of Assessment declared that classroom (and large-scale) assessments should support student learning and success by integrating assessments as part of the learning process rather than maintaining assessment as a separate goal (Chudowsky and Pellegrino 2003; NRC 2001).

Kindergarten entry assessments (KEA) have been devised to evaluate young children around the time of school entry—right before kindergarten, during, or after kindergarten entry—and have multiple purposes: to monitor readiness, inform instruction, monitor education trends over time, and/or provide indicators of high-stakes accountability (Maxwell and Clifford 2004). As of 2021, 38 states in the US now require schools to complete readiness assessments to determine children’s level of development prior to entering kindergarten (Yun et al. 2021). However, there seems to be a dearth of well-validated KEAs to appraise young children’s skill levels and learning over time. Concerns have been raised by child development experts that some assessments focus narrowly on reading or math to the detriment of other domains such as the sciences, social studies, music, and arts (Yun et al. 2021). This limited perspective can lead to teachers favoring academics in their teaching over other domains to teach to the test, or to favoring teacher-led instruction rather than active, experience- or play-based approaches to learning (Yun et al. 2021).

The National Association for the Education of Young Children (NAEYC) has made detailed recommendations for developmentally appropriate practice (DAP), including that both formative (measuring progress) and summative (at specified endpoints) assessments are needed to best support children’s early learning experiences. Below, we briefly describe for comparison some KEAs that are well-known and commonly used, many of which are technology-based, like BELLA (drawn from Yun et al. 2021).

### 2.3. A Few Examples of Well-Known KEAs

Teaching Strategies (TS-GOLD; Teaching Strategies LLC 2022) is one of the most commonly used KEAs in the USA. It is a formative, observation-based assessment for infants through children in the 3rd grade. The differentiating feature of this tool is the ongoing nature of the assessment process: it is carried out by teachers during regular, everyday activities on a continuous basis. It guides teachers to rate their students on 38 objectives across 11 domains (Social and Emotional, Physical, Language, Cognitive, Literacy, Mathematics, Science and Technology, Social Studies, the Arts, and English Language Acquisition). The cognitive skills addressed by TS-GOLD include attention, persistence, problem-solving, curiosity, motivation, flexibility, and inventiveness (grouped as “positive approaches to learning”); memory and associative thinking (“remembers and connects experiences”); classification; and symbolic and imaginative thinking (“uses symbols and images to represent something not present”) (Teaching Strategies LLC 2022). TS-GOLD also asks teachers to evaluate children’s social–emotional skills (Teaching Strategies LLC 2022). TS-GOLD attends to several important skills that reflect a whole-child developmental perspective in early childhood education. However, it may be challenging for teachers to observe, record, and evaluate these actions in their students daily.

Similarly teacher-led, the Brigance Early Childhood Screens III (French 2013; Yun et al. 2021) is a set of developmental screeners that address children’s development from infancy through 1st grade. It is brief, including only 13 items, but covers three domains at the Kindergarten/1st-grade levels: academic skills and cognitive development (literacy and mathematics), language development (receptive and expressive), and physical development (gross and fine motor). Each item is a performance task delivered one-on-one and scored by a teacher. The tasks vary according to the targeted skill (e.g., standing on one foot for 1 s with eyes closed, reading uppercase letters). Materials for teachers evaluating Spanish-speaking children are provided. As a screening tool, Brigance is focused on evaluating children for particular milestones so that early delays can be addressed. Like TS-GOLD, no part of Brigance involves a computerized interface for the children.

In contrast, one direct assessment delivered via a tablet commonly used within early educational settings is the Standardized Test for the Assessment of Reading-Early Literacy Enterprise, STAR-EL (Shapiro and Gebhardt 2012). STAR-EL is a computerized adaptive assessment that targets early literacy and numeracy (word knowledge and skills, comprehension strategies, and numbers and operations) in preschool through grade 3. It is designed to be taken independently by a child on a computer or tablet. STAR-EL is an example of a contemporary assessment based on traditional approaches, including multiple-choice items that cover basic academic areas. It does not target any cognitive skills; it is available in Spanish. STAR-EL is progressive in its tablet-based delivery; however, it does not appear to make full use of tablet-based technology (i.e., capitalizing on different item formats or the availability of engaging graphics). See Supplementary Materials for a summary of this comparative information, Table S1.

## 3. BELLA and Its Contributions to the Development and Evaluation of Cognitive Skills

BELLA was designed to enhance the assessment of preschool children by combining child-friendly, enjoyable activities with state-of-the-art methodology in technology-based testing. BELLA assesses aspects of early literacy, numeracy, science, and social-emotional development along with a set of cognitive skills (analytical, creative, and practical thinking). It is aligned with the theoretical frameworks of the USA national education standards (Department of Health and Human Services 2010; U.S. Department of Education 2015) as well as the Texas Essential Knowledge and Skills (TEKS) guidelines (Texas Education Agency 2022), yet addresses common standards across several states. BELLA’s items (over 700) can comprise summative assessments to gauge preschoolers’ proficiency in domains relevant to school readiness. BELLA also includes novel items that are particularly well-suited to emphasize the development of children’s reasoning and creativity, both of which are considered important 21st century skill domains.

### 3.1. BELLA’s Knowledge Domains

#### 3.1.1. Early Literacy Skills 

Early literacy skills are the precursors of reading and writing (Dickinson and McCabe 2001). The skills most predictive of later performance are phonological awareness, vocabulary, oral expression, and print knowledge (Connor et al. 2006; Lonigan et al. 2000). In ELL bilingual populations, phonological awareness, along with vocabulary and letter knowledge, is one of the main precursors to literacy (Lonigan et al. 2013; National Report Panel 2000). BELLA assesses three: phonological awareness, print knowledge, and letter-sound correspondence.

#### 3.1.2. Early Numeracy Skills

A wealth of evidence has shown that children’s informal knowledge must be established before more formal aspects of mathematics are introduced and that children’s acquisition of informal knowledge varies due to differences in their exposure to numerical vocabulary and concepts in the home (Siegler and Lortie-Forgues 2014). Thus, BELLA‘s easiest (as per children’s normative developmental progress) early numeracy items (i.e., targeting basic proficiency levels according to curricular standards) are simple, non-verbal tasks working with quantities of less than 10. These are followed by the assessment of number word and symbol recognition and counting, the early verbal precursors to formal mathematical activities (Mix et al. 2002), and the assessment of sequencing of numbered quantities (number line type activities) to ascertain children’s understanding of the correspondence between enumeration and quantity.

#### 3.1.3. Early Science Skills

Today, science education guidelines dictate that opportunities to experience scientific inquiry and exploration should begin at an early age (Lind 1999; Next Generation Science Standards 2013; Trundle and Saçkes 2015). They emphasize two aspects of early science to develop in young children: declarative knowledge (particularly in the areas of the life sciences, earth/space sciences, and physical sciences) and process skills (e.g., observing, describing, predicting, and experimenting). Children as young as three and four, it has been argued, are capable of early scientific reasoning, particularly concerning cause and effect and analogical thinking (Bullock et al. 1982; Wilkening and Sodian 2005). BELLA assesses children’s knowledge regarding living things, daily rhythms of life (e.g., time, seasons, growth), the earth and stars, and the senses. In other categories (e.g., colors, shapes, size, and measurement), BELLA assesses children’s skills in observation and inquiry in activities such as recognizing, naming, and comparing based on physical details and different ways of describing what they see.

### 3.2. BELLA’s Addition of Cognitive Skills

**Figure 1:**
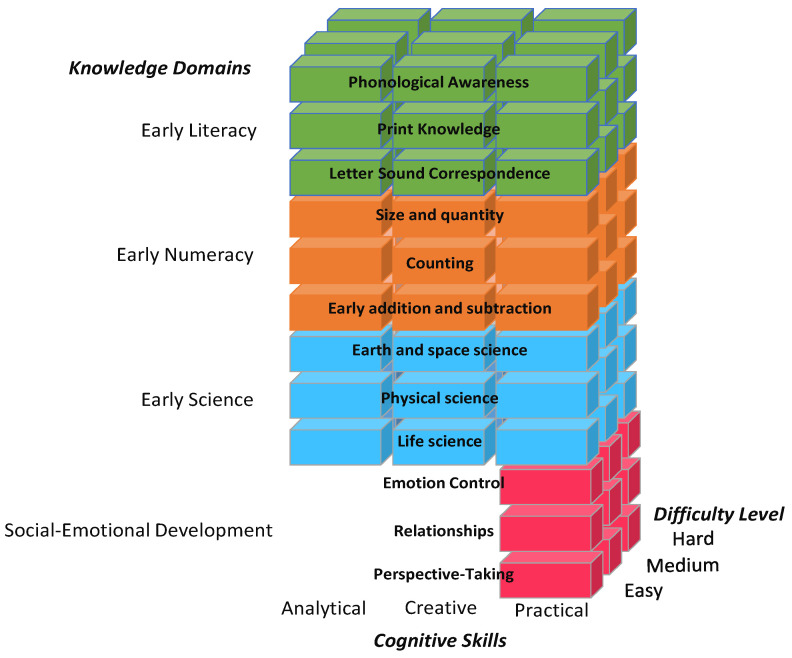
BELLA’s Structure: Knowledge Domains and Cognitive Skills. Note. Social-Emotional items are only considered practical, hence the shorter red bars.

BELLA’s items were created specifically to target knowledge domains and cognitive skills. That is, within each knowledge domain, different item types (e.g., requiring compare/contrast skills, multiple responses, the consideration of imaginative or novel situations) were designed to call upon different cognitive skills. For example, questions about how to divide cookies evenly between friends or make sure that the number of spoons matches the number of forks on the table are quantitative inquiries set within practical situations, combining numeracy within an everyday context. Figure 1 shows BELLA’s structure of targeted content knowledge and skills. The figure depicts each of BELLA’s knowledge domains: Early Literacy (green), Early Numeracy (orange), Early Science (cyan), and Social-emotional Development (magenta) and their associated sub-domains. Each domain has two dimensions: cognitive skills (analytical, creative, and practical) and difficulty level (easy, medium, hard). The social-emotional development domain has three sub-domains, three difficulty levels, but only one cognitive dimension (practical), unlike all other sub-domains. Each item in BELLA maps onto one of these combinations of domains, sub-domains, cognitive skills, and difficulty levels.

## 4. Capitalizing on Digital Technology to Capture Analytical, Creative, and Practical Skills

Throughout each knowledge domain, BELLA elicits analytical, creative, and practical thinking through innovative item content and mechanisms of item delivery. BELLA’s content is based on the knowledge components that form the foundation for the Head Start Child Development and Early Learning Framework (Department of Health and Human Services 2010), set within the cognitive framework of Sternberg’s theory of successful intelligence (Sternberg 2005, 1999). That is, BELLA’s content addresses knowledge domains and cognitive skills by assessing children’s development of early literacy, numeracy, and science while simultaneously accessing a set of cognitive skills identified as major components of general intellectual functioning. Specifically, these cognitive skills are developed in the (1) acquisition and organization of knowledge, i.e., analytical skills, such as those used in evaluating, sorting, and comparing; (2) extension or transformation of knowledge, i.e., creative skills, such as inventing, designing, or imagining; and (3) application of knowledge, i.e., practical skills, such as those used to relate, adapt and contextualize. In addition, the social-emotional skills assessed through BELLA (i.e., emotion control/emotion recognition and empathy) are central to most theoretical models of social–emotional competence in early childhood (Denham et al. 2012; Halberstadt et al. 2001; Vaughn et al. 2009). BELLA is designed to capitalize on what we know of digital media use in the context of learning and for young children. It is meant to adapt to a learning environment constantly facing innovations and new technologies. Below we briefly and selectively illustrate how some of these skills—specifically, estimation, mental flexibility, empathy, and perspective-taking—are captured in BELLA.

### 4.1. Assessing the Analytical Skill of Estimation

Quantitative estimation (from here on simply “estimation”) involves assigning a cardinal number to a discrete collection of objects or other unknown quantity (e.g., length, area, volume) without the precision of actual counting or using formal measurement tools. Estimation is a best guess or approximation based on the available information and is generally exercised when precise calculations or measurements are not possible or not necessary (Chang et al. 2011). Its importance in children’s education is twofold: first, the exercise of estimation skills is ubiquitous in everyday life, both in school and out of school, across several domains of everyday life; second, estimation skills support the development of higher level mathematical thinking and reasoning (Andrews et al. 2022; Rittle-Johnson et al. 2001; Russo et al. 2022), correlating with standardized achievement test performance as well as arithmetic and magnitude comparison (Opfer and Siegler 2007).

BELLA addresses estimation, a fundamental component of quantitative reasoning, in two sets of items. In the first type, a child is shown a long piece of cucumber alongside a slice of cucumber of a particular width. The child is asked to estimate how many more slices of about the same width (“size”) might be cut from the length of the cucumber. (Some items present a length of bread, like a baguette, instead of a cucumber.) Several measurement skills are at work here: a visual evaluation of the width of the example slice and the mental application of that width in iterations along the length of the larger piece of cucumber, which involves perceptual abilities such as spatial visualization. In effect, the child must apply one of the main estimation strategies, decomposition, in which the object being estimated is mentally decomposed into smaller units (Desli and Giakoumi 2017). To respond, children select one of four consecutive numeric choices. Then, since this is an estimate, the child is asked to provide a second possible answer. This highlights the approximate nature of estimation and introduces the notion that a single “correct” solution is not always sought; in both science and mathematics, some problems may require a reasoned guess rather than an exact answer (Jones et al. 2012). A more advanced set of estimation tasks in BELLA ask a child to consider the number of cans of paint in relation to the size of a painted area in a picture (e.g., two cans to paint a wagon), then asks the child to estimate about how many cans it might take to paint something larger or smaller (such as a toy truck or a window). Children choose from responses represented by sets of paint cans (e.g., pictures of one can, two, three, or four cans) rather than numerals and again, they are asked to choose a second possible response. While these activities may be unusual in children’s assessments, they present common, everyday problems that even the youngest children may recognize. These are, therefore, opportunities to formalize children’s quantitative understanding and reasoning.

### 4.2. Assessing Creative Skills and Flexible Thinking through Novel Tasks

Imagination is increasingly recognized as a core feature of children’s development (Harris 2000; Yogman et al. 2018), although this is not a new idea (Thibodeau-Nielsen et al. 2021; Vygotsky 1967). Imagination supports engagement in behaviors and cognitions not directly connected with one’s current reality, requiring the mental formation of places and things not sensibly present (Thibodeau-Nielsen et al. 2021; Woolley and Gilpin 2020). Imagination has been linked with many developmental outcomes, including positive peer relationships (Singer and Singer 1981, 2009), executive functioning (e.g., Carlson et al. 2014; Pierucci et al. 2014; Thibodeau et al. 2016; White and Carlson 2016), perspective-taking skills (Lillard et al. 2011), and emotion regulation (Gilpin et al. 2015). Imagination is also a key component of the creative skills needed to solve novel problems (Sternberg 1999), as exploration and evaluation can result in novel and useful outcomes (Chylińska and Gut 2020). While creativity broadly conceived is valued and prioritized in current preschool curricula, particularly in the arts, the specific cognitive skills that support creative thinking are less attended to in the classroom, although their role in academic learning has been recognized for decades (e.g., Beghetto and Kaufman 2010; Grigorenko et al. 2008; Grigorenko 2019; Runco et al. 2017; Tan and Grigorenko 2010). BELLA is designed to address this gap in two aspects: by presenting children with novel problems, and by asking them to consider that there is more than one possible “good” response to questions.

#### 4.2.1. Creative Thinking in Novel Problems

Several items in BELLA’s science domain present children with novel tasks. These questions present situations that have not occurred or could not occur in real life (e.g., houses made of chocolate, living on clouds) and, therefore, must be imagined. Children must take what they know about certain materials or phenomena and imagine how they might work in new settings, or what might result if elements could be combined in different ways. For example, one set of questions asks children to imagine, given a choice of four fruits, which two could be combined to make a new fruit that has certain properties of taste, color, or texture. The children must draw on their knowledge of common fruits and imagine the hybrids that might result when they are combined. Another set of questions asks children to combine alien-like heads and bodies that can live in specific environments (e.g., in a cave, in a tree) and eat certain foods (e.g., bugs, leaves). These types of questions are meant to stimulate playful and imaginative thinking while accessing what children might know about the natural world.

#### 4.2.2. Creative Thinking in Multiple Solutions

BELLA provides opportunities for *flexible thinking* by inviting children to provide a second plausible response to a question. In questions in the mathematics domain, the two possible responses may both be equally correct. However, in the language and science domains, one response may be better while the second is “good enough”. For example, in items that address rhyming, exact rhymes such as “cat” and “hat” may be considered exactly correct, yet “cat” and “Kate” may be considered similar enough for a poem or some other literary circumstance. Classroom conventions generally emphasize that there is only a single “correct” response to any question. By departing from this notion, divergent thinking is encouraged, in which multiple ideas are produced, some being more pertinent than others. This deviates from the mindset that a single correct answer is the best response to a problem.

### 4.3. Assessing the Practical/Social Skills

During early childhood, the most practical skills for children include those within the domain of social-emotional development—that is, in dealings with the self (e.g., self-regulation, attention), situations (e.g., social problem solving), and others (e.g., prosocial behavior, emotion recognition). Contemporary theoretical models conceptualize social-emotional competence in early childhood as a multidimensional construct that includes skills such as emotional knowledge (e.g., expression knowledge and situational knowledge), self-regulation (e.g., managing emotions, cognition, and behavior), self-awareness (e.g., identifying emotions), social awareness (e.g., perspective taking) as well as relational and prosocial skills (e.g., social problem solving, cooperation, seeking help) (Denham et al. 2012; Halberstadt et al. 2001; Vaughn et al. 2009). Social and emotional skills are increasingly recognized as crucial for children’s success in school, as well as in other settings (Darling-Churchill and Lippman 2016). In early preschool, children interact with peers frequently, during which emotional expressions and arousal must be apprehended and managed, specific friendships are formed, and prosocial behaviors and interactions emerge (Denham et al. 2009). Recent literature has emphasized the need for accurate and psychometrically sound assessments of social-emotional skills in early childhood that can be administered on a continuous basis, particularly for ELL, whose language and particular socialization processes may not be addressed in English-based assessments (Darling-Churchill and Lippman 2016; Denham et al. 2009; Snow 2011). BELLA supports assessing early social-emotional skills in three domains: emotion control/emotion recognition, emotion control/relationships, and empathy.

#### 4.3.1. Emotion Control/Emotion Recognition

Emotion control is an aspect of self-regulation, along with behavior control and the control of one’s attention (Gagne et al. 2021). Emotion-related self-control processes include those involved in managing the “cool” or conscious aspect of emotional response, as well as the involuntary or “hot” reactions to situations (Eisenberg et al. 2010). Part of emotion management is identifying feelings, both in oneself and in others (Izard et al. 2001). BELLA includes several items tapping basic emotion recognition, such as items that ask a child to identify basic facial expressions. In these items, children are shown four faces and instructed, for example, to: “Look at these faces. Touch the one that is happy”. Six cross-culturally validated facial expressions of emotion are targeted: happy, sad, angry, afraid, surprised, and disgusted.

#### 4.3.2. Emotion Control/Relationships

A second important aspect of emotional control is the recognition and management of emotions that emerge in response to unexpected events or experiences (Eisenberg et al. 2010). To assess skills in this area, children are presented with a situation in which something unexpected is encountered by someone, and they are asked how that character feels. For example, “Bird’s favorite color is blue. He saw that there were blue balloons at his birthday party. How did Bird feel?” Children choose an answer by selecting an emoji-like face with the corresponding facial expression. In these items that require more advanced emotion processing skills, including self-other differentiation, children show their understanding of emotions as responses to things that happen (Gagne et al. 2021).

#### 4.3.3. Empathy and Perspective-Taking

Empathy has been defined as an affective state that arises when a person perceives the emotions of another and experiences congruent emotions (Eisenberg and Miller 1987). It is well-known that social–emotional skills, such as emotion matching or the ability to experience emotions vicariously, begin to emerge in early childhood (Eisenberg and Lennon 1980; Eisenberg and Miller 1987). A cognitive component of empathy involves the mental processes of perceiving, recognizing, and understanding the internal state of another person to the point of taking on that person’s role or perspective (Iannotti 1975). The affective component of empathy involves the emotional response (conscious or unconscious) of the observer upon perceiving the internal state (emotions, thoughts, or attitudes) of the other person (Zoll and Enz 2010). BELLA items access the cognitive component of empathy by asking children to consider a social situation and report how the character within the situation is feeling. BELLA’s cognitive empathy items ask a child to consider a social situation and indicate how someone in this situation might feel. For example: “Crocodile is having friends over to play. He likes his friends. How will he feel when they come over?” These items particularly address children’s ability to comprehend social situations and associate them with the emotions they might elicit.

## 5. BELLA’s Psychometric Properties with Regard to Cognitive Skill Assessment

Data were collected to preliminarily evaluate BELLA’s psychometric performance in both knowledge and cognitive domains. We describe this study here.

### 5.1. Method

#### 5.1.1. Sample

Data for the present study were collected across 17 schools from 26 March 2019 to 31 August 2022. Due to constraints imposed by the COVID-19 pandemic, most of these schools were private preschools. The final dataset includes 506 children ages 3–6 years old (there were a few six-year-olds in our participating preschool classrooms).

#### 5.1.2. Procedure

For about half of the sample, BELLA was administered by trained research assistants who worked with children at the school sites. In these cases, sessions with BELLA were conducted in segregated spaces to minimize distractions for the children. For the rest of the sample, researchers trained schoolteachers in administering BELLA. Teachers then carried out sessions with BELLA in quiet classrooms in the morning, before the official start of school, or during group activity time. In all cases, teachers or research assistants worked one-on-one with each child as they engaged with BELLA. Each session with BELLA consisted of 33 items, taking 20–30 min. Most children engaged with BELLA once or twice. A small percentage of children engaged with BELLA three or more times (see Table 1). Each session included distinct sets of parallel items across domains and difficulty levels.

#### 5.1.3. Data Analyses

For each domain of BELLA, two types of test validation metrics were estimated per item: pass rates and discrimination. Pass rates were the number of correct responses for each item. Discrimination rates were calculated using the extreme group method, which involves partitioning participants into four groups based on their score percentiles and then calculating the difference between the means of the lower and upper quartiles of scores. Discrimination was estimated for items grouped by cognitive skill, domain, subdomain, and difficulty. Prior to these analyses, items were grouped based on scoring types. Partial credit items (*n* = 62) are awarded 34, 50, or 66 out of 100 points. These items ask children to provide more than one correct or acceptable response; in some cases, one response is more accurate. Non-partial credit items (*n* = 142) are binary, correct, or incorrect, awarded either 0 or 100 points. Here we share results regarding the evaluation of cognitive skills by BELLA.

### 5.2. Results

Table 2 presents their demographic descriptive statistics, as well as the number of times children engaged with BELLA over the course of the study.

Item difficulty by item types, based on pass rates, showed that a majority of BELLA’s items presented acceptable levels of difficulty (see Table 2), being neither too easy (>90% correct responses) nor too difficult (<10% correct responses). Pass rates for partial credit items are calculated as the mean score across items.

Table 3 presents the item discrimination distributions for partial and binary items by item type and cognitive skills by and across skill domains.

**Figure 2:**
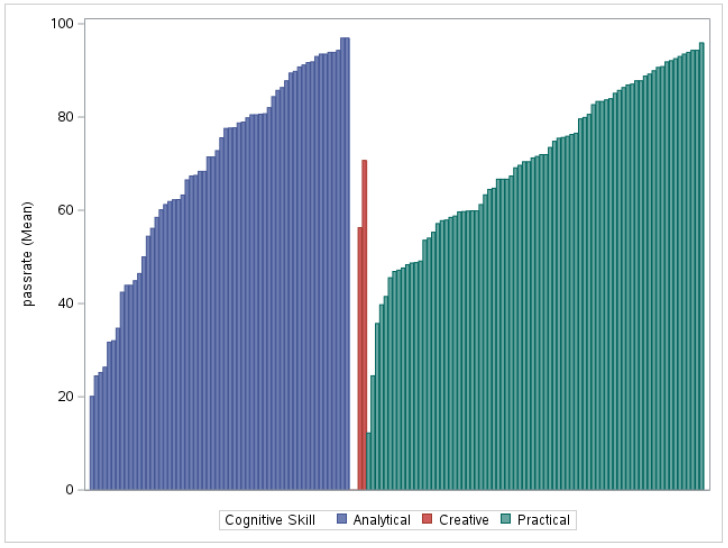
Pass Rates for Binary Items by Cognitive Skill.

**Figure 3:**
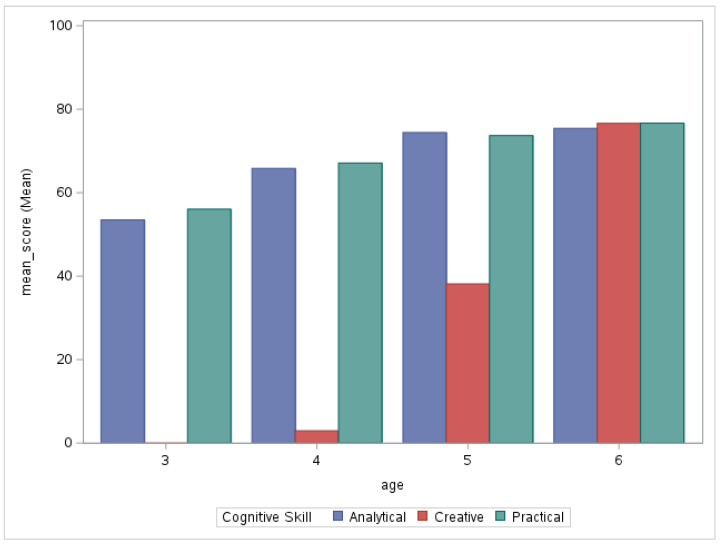
Pass Rate Means for Binary Items by Cognitive Skill by Age.

The calculated values were all positive, indicating that the high-performing group of students in the current sample is always more efficient than the lower-performing groups, with binary discrimination means falling into the 0.3–0.5 range and partial credit items trending slightly lower, in the 0.2–0.3 range. In binary items, cognitive skill discrimination seemed to differentiate more in the creative cognitive skill, while partial credit items mostly belonged to a single cognitive skill domain (i.e., creative cognitive skill), precluding an analysis of differences across cognitive skill domains. The highest discrimination values by knowledge domain were found in mathematics for binary items and in science for partial credit items. According to the average item discrimination values for the cognitive skill domains, as indicated in binary items, the creative skill items performed slightly better. All partial credit items were primarily employed to assess creative cognitive skills. Figure 2 and Figure 3 illustrate the item-level pass rates for cognitive skills across all items and by age, respectively, as indicated in the binary items. The item level pass rates indicate that pass rates in scores for analytical and practical skills increased with age, along with a marked improvement in creative items. There were few obvious outliers in all cognitive skill categories.

As these data indicate, BELLA’s items were able to differentiate children’s skill levels in our targeted cognitive domains to some extent, with creative items doing this particularly effectively.

## 6. Discussion & Conclusions

While the educational sector at large is in agreement that teaching and learning in the current era must move away from emphases on memorized facts and declarative knowledge toward skills that can address an increasingly dynamic world, the capability to formally assess these skills, particularly in the early years, is still evolving (Care et al. 2018; Soland et al. 2013). We have argued here that a broad range of cognitive skills important for the 21st century context may be assessed and fostered in early childhood. BELLA achieves this through questions specifically designed to invite basic forms of reasoning, such as comparing and recognizing patterns, flexibility, imagination, and open-mindedness, and the application of these in everyday situations. While the learning progression of these and other 21st century skills are still being mapped out to guide educational settings (Care et al. 2018), BELLA represents an early effort to integrate preschool academic content with skill development.

The initial results of administering BELLA items to preschool children illustrate that such assessment is possible while simultaneously evaluating children’s developing academic knowledge. Further, BELLA illustrates the capability of innovative items presented in digital formats to evaluate and distinguish across ages children’s capability to cope with novel problems successfully, as indicated by the higher discrimination of BELLA’s creative items.

Recognizing and encouraging a diverse range of skills among diverse child subpopulations is a step toward the diversity, equity, and inclusion we hope to see in all spheres of activity where big picture problem-solving needs to occur. It is also part of a movement toward individual and community empowerment through developing a broad set of life skills that include empathy, participation, creativity, communication, and self-awareness (UNICEF 2019). In this article, we have introduced a new assessment tool, BELLA, that intends to make inroads toward diversity starting at the earliest stages of formal education, where a variety of cognitive skills can be both identified and fostered, leading to a greater diversity of problem-solvers down the road. Our recognition of the new geologic era of the Anthropocene and the urgent global problems that accompany it demands a renewed focus on developing the cognitive skills needed for solving novel problems.

## Figures and Tables

**Table 1:** Descriptive Statistics for the Study Sample (*N* = 506).

	*n*	Proportion
Age		
3	85	16.80%
4	233	46.05%
5	123	24.30%
6	65	12.85%
Female	218	43.08%
Number of BELLA sessions		
1	200	39.53%
2	219	43.28%
3	69	13.67%
>3	18	3.56%

Note. Each session with BELLA presented different items to children.

**Table 2:** Difficulty of Partial Credit and Binary Items by Mean Pass Rate.

Item Type	Difficulty	Item Count	%
Partial Credit	Too easy (>90)	1	1.61%
	Acceptable (10–90)	58	93.55%
	Too hard (<10)	3	4.84%
Binary	Too easy (>90)	23	16.20%
	Acceptable (10–90)	117	82.39%
	Too hard (<10)	2	1.41%

**Table 3:** Descriptive Statistics For Discrimination Values By Item Type, Cognitive Skill, and Academic Domain.

Item Types	Number of Items	Mean	SD	Minimum	Maximum
Partial Credit Items					
Overall Discrimination	62	0.2507	0.1328	−0.05	0.5476
Analytical	2	0	0	0	0
Creative	60	0.259	0.1266	−0.05	0.5476
Literacy	14	0.2194	0.1324	0	0.44
Mathematics	12	0.2438	0.1537	−0.05	0.4231
Science	36	0.2652	0.1272	0.0171	0.5476
Binary Items					
Overall Discrimination	142	0.3543	0.159	0	0.88
Analytical	60	0.3424	0.1493	0.0286	0.7424
Creative	4	0.4396	0.5077	0	0.8889
Practical	78	0.359	0.1367	0.0572	0.7024
Literacy	24	0.3422	0.1148	0.1064	0.5294
Mathematics	28	0.3815	0.225	0	0.8889
Science	72	0.3466	0.1522	0.0286	0.7424
Social/Emotional	18	0.3587	0.1147	0.1765	0.5238

## Data Availability

Data can be obtained upon request from the corresponding author.

## Supplementary Materials

The following supporting information can be downloaded at: https://www.mdpi.com/article/10.3390/jintelligence11070143/s1, Table S1. A comparative summary of the academic and cognitive skill domains of commonly used KEAs.
