# Supplementary material for: Assessing Cognitive Skills in Early Childhood Education Using a Bilingual Early Language Learner Assessment Tool
Source: J Intell. 2023 Jul 17;11(7):143. doi: 10.3390/jintelligence11070143 (PMC10381262; doi:10.3390/jintelligence11070143)
Supplement: Supplementary file 1 [file jintelligence-11-00143-s001.zip › jintelligence-2300870-supplementary.pdf]

Table S1.

A comparative summary of the academic and cognitive skill domains of commonly used KEAs

		BELLA	TS-GOLD	Brigance	STAR-EL
	Administration<br>Delivery<br>Item formats	Teacher-led<br>Tablet<br>Various	Teacher-led<br>Observation<br>Various	Teacher-led<br>Teacher<br>Various	Independent<br>Computer/Tablet<br>Multiple Choice
Academic Domains	Early literacy	x	x	x	x
	Early numeracy	x	x	x	x
	Early science	x	x		
Cognitive Skill Domains		Analytical<br>Creative<br>Practical<br>Social- emotional	Social emotional<br>Attention<br>Persistence<br>Problem-solving<br>Curiosity<br>Motivation<br>Flexibility<br>Inventiveness		
Other Domains			Social studies<br>Arts<br>English Language acquisition<br>Physical development	Physical development	
